# PVC.js: visualizing C programs on web browsers for novices

**DOI:** 10.1016/j.heliyon.2020.e03806

**Published:** 2020-04-23

**Authors:** Ryosuke Ishizue, Kazunori Sakamoto, Hironori Washizaki, Yoshiaki Fukazawa

**Affiliations:** a Department of Science and Engineering, Waseda University, Tokyo, Japan; b National Institute of Informatics, Tokyo, Japan; c WillBooster Inc., Tokyo, Japan; d SYSTEM INFORMATION CO.,LTD., Tokyo, Japan; e eXmotion Co., Ltd., Tokyo, Japan

**Keywords:** Science, C language, Browser application, Visualization

## Abstract

Many researchers have proposed program visualization tools for memory management. Examples include state-of-the-art tools for C languages such as SeeC and Python Tutor (PT). However, three problems hinder the use of these and other tools: capability (P1), installability (P2), and usability (P3). (P1) Tools do not fully support dynamic memory allocation or File Input / Output (I/O) and Standard Input. (P2) Novice programmers often have difficulty installing SeeC due to its dependence on Clang and setting up an offline environment that uses PT. (P3) Revisualization of the modified source code in SeeC requires several steps. To alleviate these issues, we propose a new visualization tool called PlayVisualizerC.js (PVC.js). PVC.js, which is designed for novice C language programmers to provide solutions (S1–3) for P1–3. S1 offers complete support for dynamic memory allocation, standard I/O, and file I/O. S2 involves installation in a user web browser. This system is composed of JavaScript programs, including C language execution functions. S3 reduces the steps required for revisualization. To evaluate PVC.js, we conducted two experiments. The first experiment found that students using PVC solved a set of four programming tasks on average 1.7—times faster and with 19% more correct answers than those using SeeC. The second experiment found that PVC.js has a visualization performance equivalent to PT, and that PVC.js is more effective than existing general debugging tools for novices to understand programs in cases where the values of important variables change and the control flow is complicated.

## 1 Introduction

Various visualization techniques have been proposed to aid programmers in understanding the program execution status [1], [2], [3], [4], [5].^1^ Most existing debuggers and integrated development environments such as GDB and Eclipse provide limited features to visualize the program execution status. Typically, these applications display simple text outputs, but do not visualize the relationships between variables, pointers, and memory. Learning how to use these tools is often difficult for novice programmers (hereafter referred to as novices).^2^ Instead of enhancing the understanding of programming languages, these tools often hinder novices.

C programming language (C language) is popular and is typically one of the first languages learned by novices. However, mastery of C language requires that users learn a basic but difficult-to-grasp concept of memory management, including pointers and dynamic memory allocation. This can be extremely challenging for novices [8], [9], [10].

To assist novices, previous studies have proposed tools to visualize the program execution status [11], [12], [13], [14]. For example, SeeC and Python Tutor (PT) are state-of-the-art tools that effectively visualize C programs. However, three problems affect these and other tools: **capability (P1), installability (P2), and usability (P3). (P1)** SeeC does not fully support dynamic memory allocation. It displays the size of the allocated memory in bytes, but omits more detailed memory values. PT does not support file Input / Output (I/O) and standard Input. **(P2)** It is difficult for novices to install SeeC due to its dependency on Clang, a compiler. Moreover, students struggle to use PT in an offline environment, and teachers must setup PT for C language on their computer. **(P3)** SeeC is a desktop application consisting of a compiler and a visualizer. Users cannot modify the source code during SeeC's visualization. Thus, SeeC requires many steps to modify and revisualize C programs. PT does not have this problem because it is a web application running on a browser window.

To solve **P1–3**, we propose PlayVisualizerC.js (PVC.js). PVC.js is an interpreter of C languages and has features to visualize the program execution status. It provides three solutions **(S1–3)** to the aforementioned problems. **(S1)** PVC.js can fully visualize variables, pointers, arrays, dynamically allocated memory, and their relationships. It supports standard I/O and file I/O. **(S2)** PVC.js is implemented as a JavaScript application that works in a browser without a web server. Thus, installation requires only a browser. It does not require a web server or an online environment. **(S3)** PVC.js allows users to revisualize a program after source code modifications using a single button click.

The novel contributions of this paper are as follows:

1) PVC.js can help novices understand the program execution status and behaviors.
2) PVC.js can be used immediately after downloading from https://github.com/RYOSKATE/PlayVisualizerC.js.
3) We conducted an experiment and a questionnaire to verify that PVC.js addresses **P1–3**.

## 2 Related works

Previous studies have indicated that the concepts of memory and pointer are extremely challenging for novices. For example, Lahtinen et al. investigated the difficulties faced by novices in learning programming languages [10] and found that more than 500 students indicated that finding bugs in a program is the most difficult part of learning to program. Moreover, pointers and references are identified as two of the hardest programming concepts.

Many studies have proposed visualization techniques and tools, which can effectively aid novices' understanding of these programming concepts. Sorva et al. surveyed program visualization systems and tools to teach beginners about the runtime behaviors of computer programs [15]. They reported that software visualization can be divided into Program Visualization (PV) and Algorithm Visualization (AV). Furthermore, PV can also be roughly subdivided into visualization of static structures and visualization of runtime dynamics. According to their definition, our research is a visualization of the runtime dynamic.

Tools that act as debuggers are often used to visualize the runtime dynamics. Debuggers were originally used by programmers to find and remove bugs. However, educators and researchers found that debuggers can help novices learn, and they began to use them as part of classroom lessons. Cross et al. explored the use of an integrated debugger as a tool to aid the understanding of novice Java programming students in CS1 [16]. Then they developed a tool called jGRASP [17], [18], [19]. jGRASP is an Integrated Development Environment (IDE) that supports the visualization of data structure. It can generate control a structure diagram (CSD) of C and Java languages.

Some research has focused on visualizing the memory state of C language. Koike and Go proposed SuZMe, which visualizes the memory state by byte unit with a horizontal straight line [12]. SuZMe also checks the values of variables and memory allocation in detail. Milne et al. proposed a program visualization tool named OGRE [1]. OGRE generates planes to represent memory space such as Global, Heap, main(), and function(). An object is represented as a figure within three-dimensional space and cylinders connecting figures represent references. Egan and McDonald proposed SeeC, which visualizes the running state of a C program [11]. Moreno et al. proposed Eliot, which is an interactive animation environment to visualize algorithms written in the C programming language [20].

Similar to our efforts for PVC.js, previous studies have developed these tools as web applications to improve accessibility. One example is Python Tutor (PT), which is a program visualization tool proposed by Guo that specializes in supporting Python and the embeddability in web-pages [21], [22]. Currently, PT supports visualization of other languages, including C language. Our literature investigation revealed that SeeC and PT are currently the most useful and widely available state-of-the-art tools for novices to learn about memory management in C language. In addition, other studies have investigated non-C languages in the field of program visualization research.

Milne and Rowe also investigated challenges surrounding learning and teaching of object-oriented programming (OOP) [9]. Their questionnaire showed that learning copy constructor and virtual function is difficult, primarily due to the fact that memory and pointer are hard concepts to grasp. These studies reveal that understanding memory and pointer are important steps towards progressing in programming ability.

Other tools such as Jeliot [23] and OOP-anime [2] support OOP languages such as Java. These tools show the relationship between class, instance, and variable with figures such as lines and boxes.

These studies seem to visualize the same concept as C memory management and pointer reference. This suggests that proposing such tools to aid in learning and understanding of the concept is worthwhile and should not be limited to a specific language.

## 3 Problems with state-of-the-art tools

**Figure 1:**
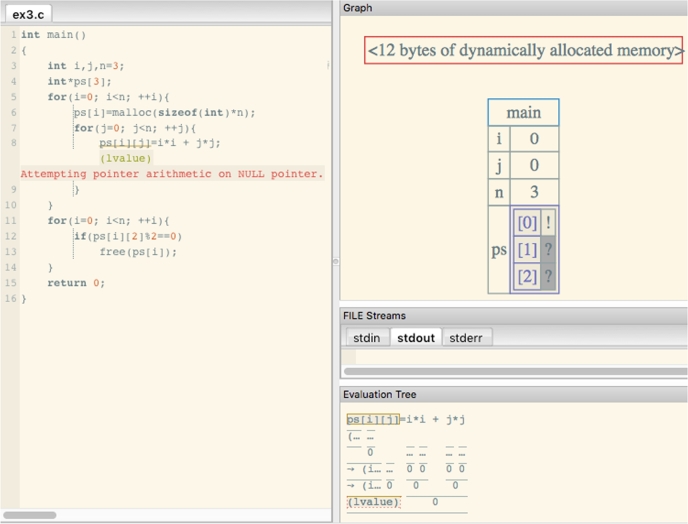
Screenshot of SeeC.

**Figure 2:**
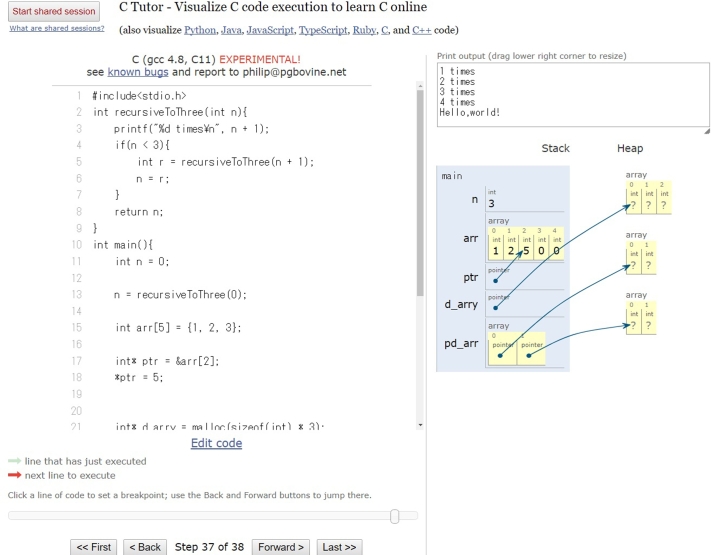
Screenshot of PT.

Fig. 1 and 2 show the visualization results of SeeC and PT, respectively. The effectiveness of these two visualization expressions are similar. They have three common problems: **capability (P1), installability (P2), and usability (P3).** Table 1 summarizes these programs and compares the features of PVC.js, SeeC, and PT.

**Table 1:** Comparison of PVC.js, SeeC, and PT functionalities.

Comparison items	PVC	SeeC	PT
**Summary**			

Capability (P1)	OK	(NG) It does not support malloc.	(NG) It does not support I/O.
Installability for client (P2)	(OK) Only a browser	(NG) Difficult to setup	(OK) Only a browser
Installability for server (P2)	(OK) Not required (or just place HTML files)	(NG) Not supported (Desktop application)	(NG) Very difficult to setup
Usability (P3)	(OK) Open in a browser, write the code, and press execute button.	(NG) Compiling is necessary beforehand. Visualizer cannot edit the code.	(OK) Open in a browser, write the code, and press execute button.

**Language feature support**			

malloc	Y	N	Y
I/O	Y	Y	N

**Visualization expression**			

Variables grouped by stack	Y	Y	
Pointer references represented by arrows	Y	Y	Y
Addresses of variables shown	Y	N	N
Display values of uninitialized variables	Random number	! or ? mark	An emoji
Visualization method	Run and create trace data using a proprietary execution environment (Junicoen).	Create execution trace data using LLVM (Clang).	Create execution trace data using GCC and a hacked Valgrind.

**Capability (P1)**: SeeC does not support dynamic memory allocation (e.g., malloc) completely. Instead it shows the byte size of dynamically allocated memory in text. Fig. 1 illustrates an example of a visualization result from SeeC, which includes only the line < *n bytes of dynamically allocated memory* >. As this example shows, SeeC users cannot see the content of the memory allocated by programs such as *malloc*. On the other hand, PT does not allow file I/O and standard Input due to security issues. It is inconvenient that these functions cannot be used.

**Installability (P2)**: The dependency of SeeC on Clang makes installation somewhat difficult. Many novice-oriented visualization tools proposed in previous studies require users to setup an execution environment. This is the main obstacle preventing users from installing these kinds of tools, regardless of a tool's usefulness [24]. Moreover, these tools are usually restricted to specific operating systems.

Another problem with PT is installability on a server computer. Even if students or teachers try to use PT, setting up a server program for C language on their own computer can be burdensome.^3^

For these reasons, SeeC and PT are insufficient for novices to write and visualize their programs in class.

**Usability (P3)**: Users cannot modify source code during SeeC's visualization because the source code is shown in a read-only text area. To revisualize a program, SeeC requires users to execute the following four steps each time the source code is modified:

1) Modify the source code file with an external editing application.
2) Recompile to generate an executable file with SeeC on a terminal or command prompt.
3) Open the executable file to generate a recording file for program behavior and execution status visualization.
4) Open the recording file in SeeC's viewer.

PT does not have such a problem.

## 4 Overview of PVC.js

**Figure 3:**
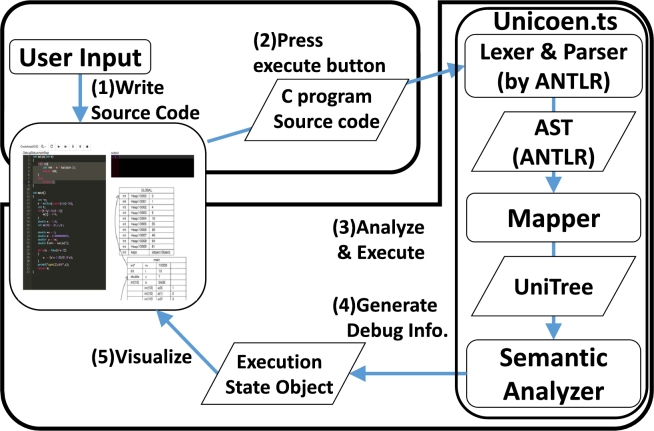
Overview of the PVC.js architecture.

**Figure 4:**
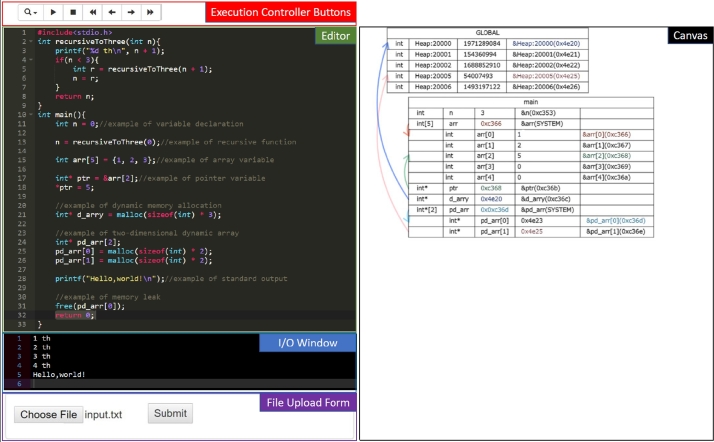
Screenshot of the PVC.js interface.

To overcome **P1–3**, we implemented PVC.js with HTML and JavaScript, including GUIs, a C parser, and a semantic analyzer. All functions work on the client side because it is developed as a JavaScript application. Fig. 3 systematically overviews the program, while Fig. 4 shows a screenshot of PVC.js. To implement our design, we used *ANTLR* to parse the source code, and *unicoen.ts* to create and execute the abstract syntax tree (AST), *UniTree*, in C languages [25]. PVC.js is a JavaScript application that simply requires users to open the html file with PVC.js in a web browser. PVC.js does not require a web server. It contains all necessary functions. Additionally, it also does not require an Internet connection. Therefore, PVC.js provides a solution **(S2)** to **installability (P2)**.

### 4.1 Tool usage

Fig. 4 shows an execution example in PVC.js. PVC.js has five GUI components: (1) editor, (2) execution controller buttons, (3) I/O window, (4) canvas for visualization, and (5) file upload form. Users can write source code in the editor. Clicking on the execution control buttons initiates the step execution. The I/O window shows the content of the standard output written by the program (e.g., *printf*) and accepts standard input (e.g., *scanf*). The canvas shows the program's execution status using tables and figures. PVC.js adaptively changes its layout to correspond with the size of the browser window.

The top of the GUI in Fig. 4 shows the program's execution controller. The controller includes the following six buttons: (i) change editor font size, (ii) initiate program execution, (iii) stop program execution, (iv) go backward for all step, (v) go backward one step, (vi) go forward one step, and (vii) go forward all steps. A statement unit executes each step.

Users can also use the local files selected from the user files form. They can use these files via programs such as *fgets, fputc*.

PVC.js has three steps:

1) Open the html file with PVC.js in a web browser.
2) Insert the source code into the editor to visualize it.
3) Press the button to execute the program. (The second button from the left in the Execution Controller Buttons initiates program execution)

Simply changing the code and pressing the execution button during visualization allows the users to change the program. Thus, PVC.js provides a solution **(S3)** to **usability (P3)**.

### 4.2 Visualization features

PVC.js, SeeC, and PT use a similar approach for visualization. The programs show values, names, and types of variables using arrows and boxes to represent pointer and stack references. For example, colored arrows and variable addresses help users understand that references differ. Movable figures make it easier to see the visualized results, while the display depth of the recursive functions informs users of how many times it has been called.

Only PVC.js supports the following important features:

• Dynamically allocated memory visualized^4^
• Memory addresses value displayed in hexadecimal
• File and standard I/O

Fig. 4 shows an example of visualization with PVC.js. There are two boxes on the canvas: main and GLOBAL. main represents a stack of the main function, while GLOBAL contains dynamic variables for heap memory allocated by malloc. The table columns in the box show (1) type, (2) name, (3) value, and (4) address of each variable. For example, the first line of main refers to a variable of *n*, which is a type *int* and has a value of 3. Moreover, some variables, such as *ptr* and d_arr, refer to other variables. The pointer reference is represented as an arrow with the same color as the reference address (e.g., the green arrow from the row containing *ptr* to the row containing arr[2]).

Figs. 1, 2 and 4 visualize the same code. Although SeeC stops its visualization, PVC.js visualizes the allocated heap memory referred to by the pointer variable $d_{a}rray$, which is not free. Therefore, PVC.js can be used to debug a program with memory leak action calls.

**Figure 5:**
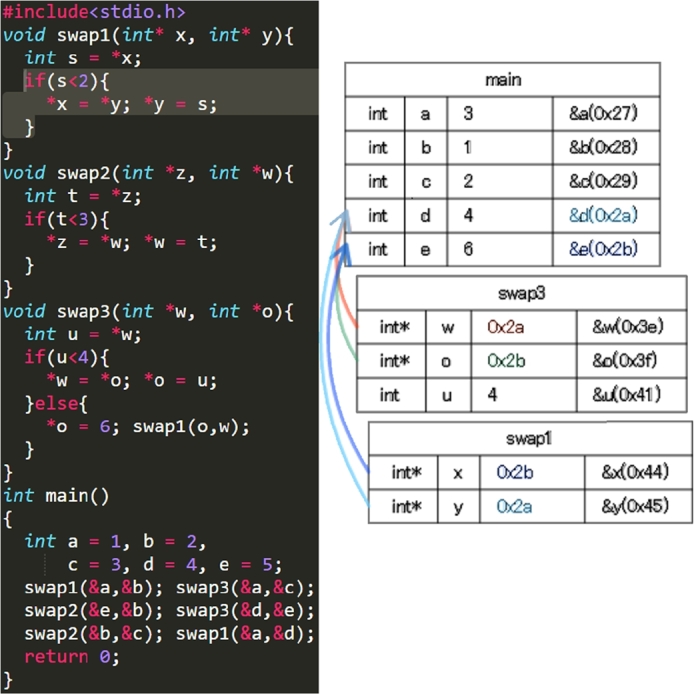
Screenshots of the sample code and the visualization results (T1): function calls with pointers.

**Figure 6:**
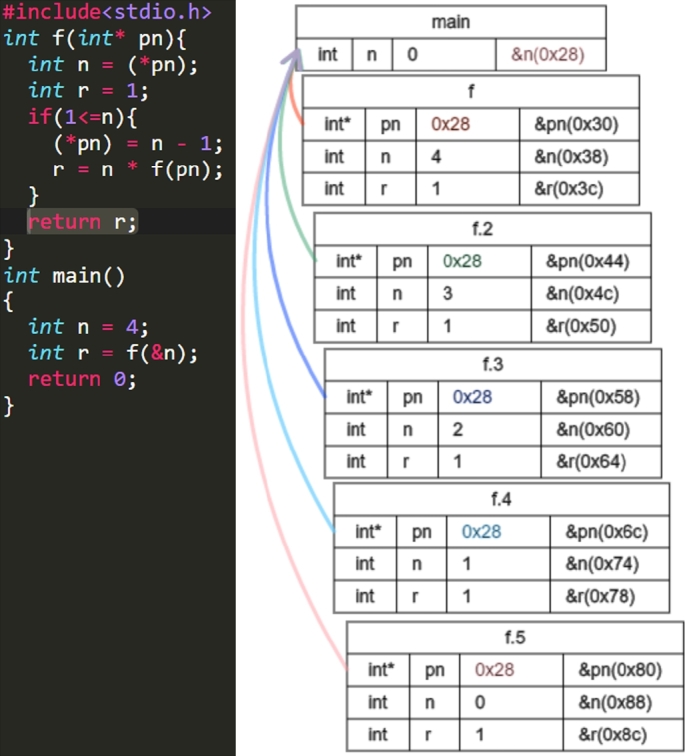
Screenshots of the sample code and visualization results (T2): recursive function calls.

**Figure 7:**
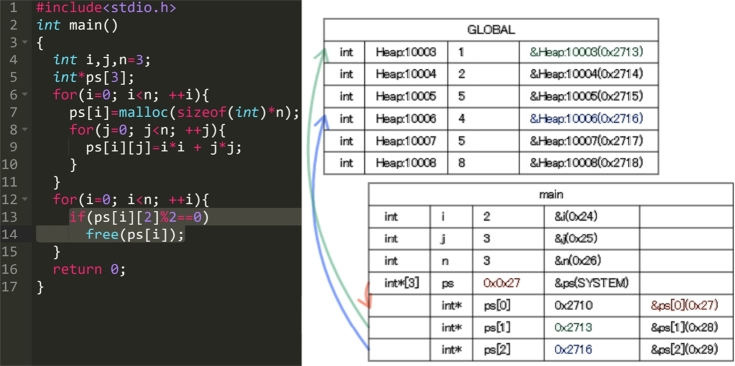
Screenshots of the sample code and visualization results (T3): dynamic memory allocations.

**Figure 8:**
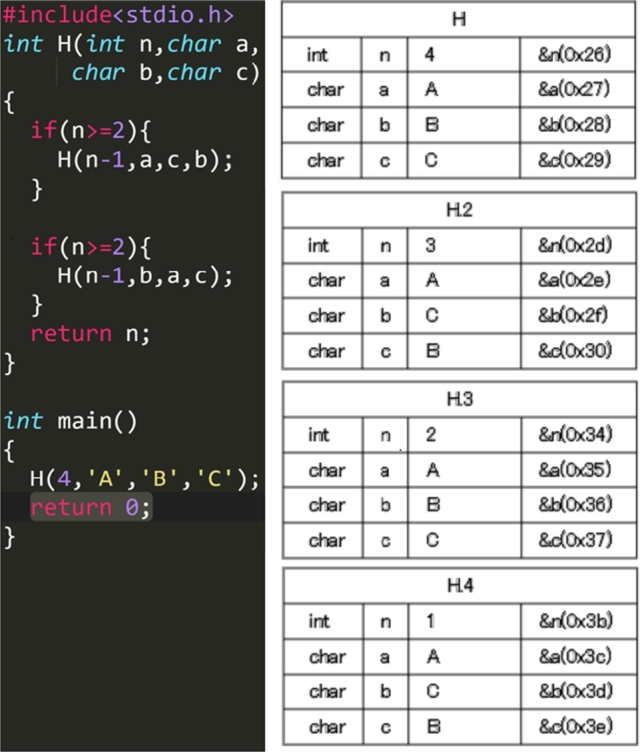
Screenshots of the sample code and visualization results (T4): complex recursive function calls.

Figs. 5, 6, 7 and 8 show additional examples. Fig. 5 shows function calls whose arguments are passed-by-pointer. Fig. 6 shows the recursive function call where the function *f* is called five times. Recall is represented in the title of each box in the form f, f.1, f.2, f.3, f.4, and f.5. Fig. 7 shows dynamic memory allocations with malloc and free. Finally, Fig. 8 shows a more complex recursive function call.

Consequently, PVC.js provides a solution **(S1)** to **capability (P1)**. Thus, PVC.js can visualize pointer and dynamic memory allocation (Fig. 4). Table 1 summarizes these solutions.

### 4.3 Language processing features

Our system does not use generic compilers like GCC or Clang or debuggers like GDB (Table 3). Generic compilers and debuggers are avoided because they are responsible for **installability (P2)**.

**Figure 9:**
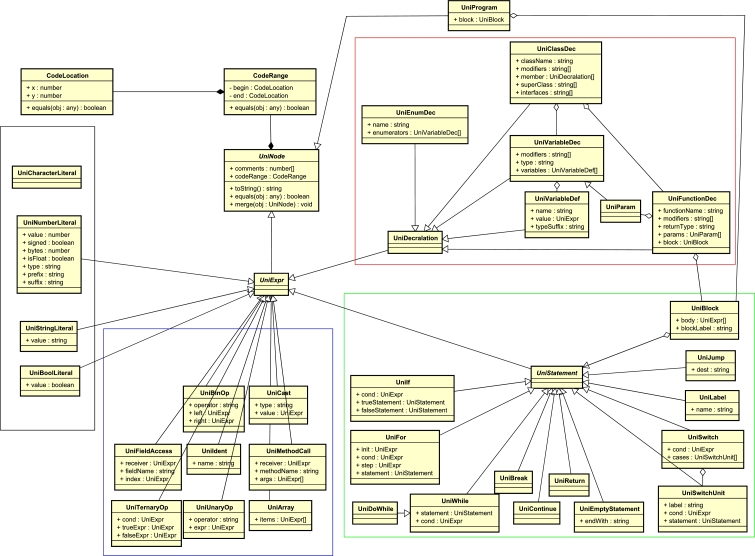
Class diagram of a UniTree in the UML.

We use *unicoen.ts*, which is a TypeScript platform that converts the source code of various programming languages into its own AST (*UniTree*). Fig. 9 shows a class diagram of a UniTree in the UML. We generated a C language parser and a lexer as well as implemented a mapper and a semantic analyzer.

The parser and the lexer are generated by *ANTLR*, which is a powerful generator that creates parser and lexer programs from the language syntax and the lexical definition files. We used the language definition file of C languages published by the ANTLR project. When a C language source code is inputted, the parser generates ANTLR's own AST, which is JavaScript object.

We implemented the mapper to plot from ANTLR's own AST to UniTree because performing semantic analysis directly using ANTLR's AST is complicated.

We also implemented the semantic analyzer to execute UniTree like a C language. This can be regarded as a simple C interpreter capable of step executions and generation of debug information.

These series of processing can be executed in a modern web browser because the transpiled TypeScript program becomes a JavaScript program.

**Figure:**
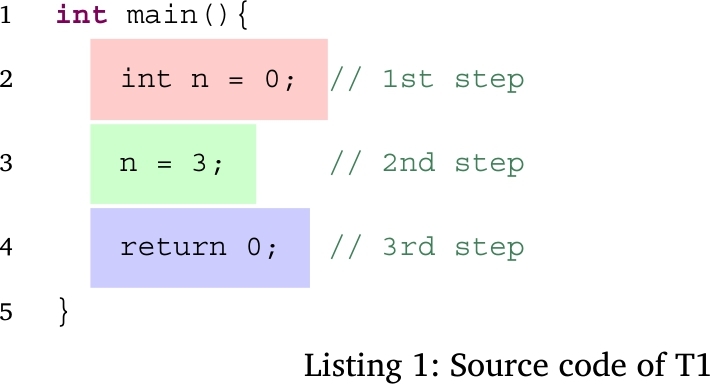

**Figure 10:**
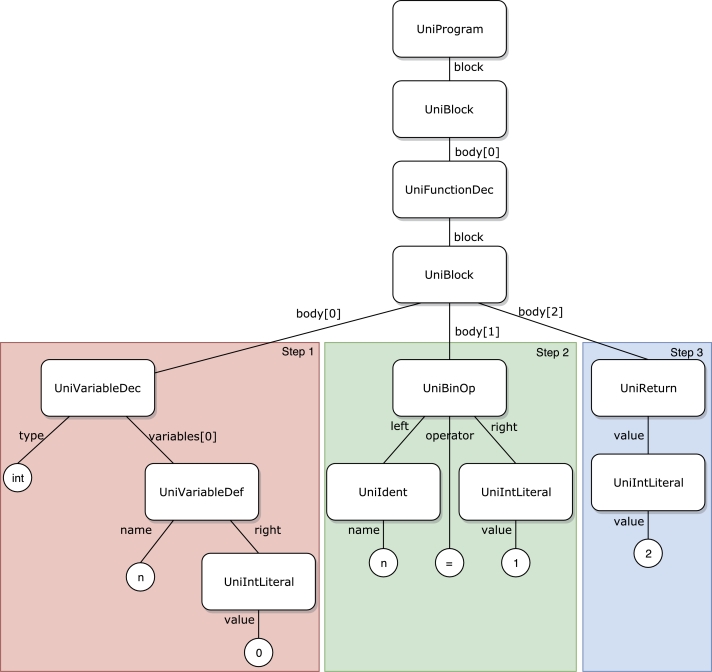
Example of a UniTree.

For example, when the source code of Listing 1 is inputted to our system, the UniTree in Fig. 10 is generated. In the first step, the semantic analyzer executes the UniTree node surrounded by a red frame like the C language. In the second step, it executes the UniTree node surrounded by a green frame. In the third step, it executes the UniTree node surrounded by a blue frame.

In our previous papers [7], language processing occurred using almost the same method in a Java program called Junicoen [25] instead of unicoen.ts. Java programs can also be used in various operating systems or environments such as browsers. We conjecture that employing a Java server locally in an offline environment is a simple but elegant solution to **installability (P2)**. However, when using PVC in an online web server, preparing a Java server on the network is burdensome for teachers. Even for use in an offline environment, students have to install Java, which is less burdensome than installing other visualization tools.

PVC.js can eliminate all of these burdens. To use in an offline environment, a user must simply open the html file with the browser used to download the PVC.js. If PVC.js is used in an online web server (e.g., to publish a customized version of PVC.js), the user simply places the PVC.js files on their web server or uses a free service hosting static webpages such as *GitHub Pages*.

Therefore, PVC.js provides a solution **(S2)** to **installability (P2)**.

## 5 Experiment

We investigated the following research questions (RQ):

• RQ1 Does PVC.js inform users of the status of the running program?
• RQ2 Does PVC.js assist novices who are learning C programming language concepts?
• RQ3 Is PVC.js a useful and more accessible application to visualize the status of a running program than similar tools?
• RQ4 How does PVC.js support the understanding of programs?

We conducted two experiments. The first experiment evaluated PVC.js with respect to RQ1–3. The second experiment also evaluated PVC.js with respect to RQ1, RQ2, and RQ4.

### 5.1 First experiment

#### 5.1.1 Experimental setting

The participants were 30 undergraduate or graduate students majoring in computer science and engineering. Ten performed the tasks using PVC,^5^ 10 using SeeC,^6^ and remaining 10 did not use a visualization tool (viewed the code only and used a pen and paper). After completing the tasks, the participants used PVC and answered a questionnaire.

The experiment involved four tasks. The source code used in this experiment is the same as the code shown on the site.^7^ Table 2 shows the examination questions (T1–4) of each task. Table 3 shows the questions (Q1–4) in the questionnaire. The participants completed the questionnaire upon finishing the tasks using PVC. Prior to the experiment, we explained PVC and provided a tutorial on how to use it. (The explanation was almost identical to that in Fig. 4.). During the experiment, we measured the time taken to answer each question and checked the answers. Q1 to Q3 were evaluated on a scale from 1 to 5, where 5 is the most positive.

**Table 2:** Examination questions (T1–4).

T1	What are the final values of variables a, b, c, d, e?
T2	What are the values of variables n, r, (*pn) when the function f is returned for a third time?
T3	What are the final values of heap memory, which has not been freed, and of the pointer variable, which refers to the memory when the main function return?
T4	When are the values of n=1, a='B', b='A', c='C'?

**Table 3:** Questions in the questionnaire about the usefulness (Q1–4).

Q1	Do you think this visualization tool helps solve the problems in the experiment?
Q2	Do you think this visualization tool is useful for C language novices?
Q3	Do you think this visualization tool is more accessible than other visualization or debugging tools (e.g., Eclipse, Visual Studio, GDB) that you have used?
Q4	In which areas of C language is this visualization program a useful learning tool? (multiple answers allowed) (A. variable, B. pointer, C. array, D. function, E. dynamic memory allocation, F. structure, G. data structures and algorithms, H. other)

#### 5.1.2 Experimental results

**Figure 11:**
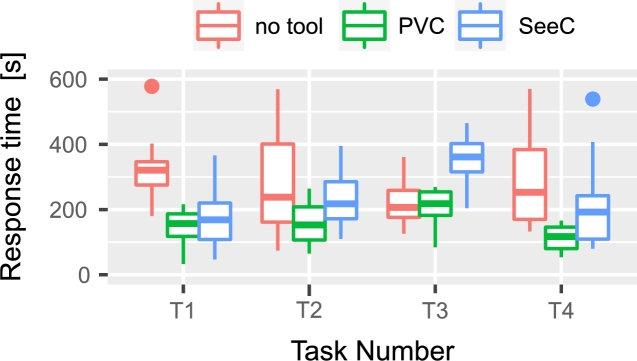
Box plot of the times required to complete each task.

**Figure 12:**
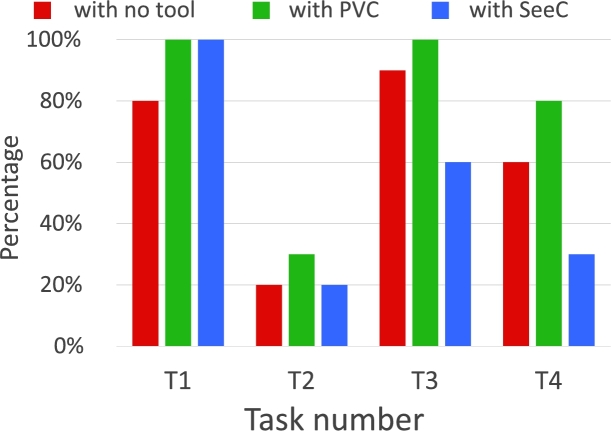
Percentages of correct answers for T1–T4 (for RQ1).

**Figure 13:**
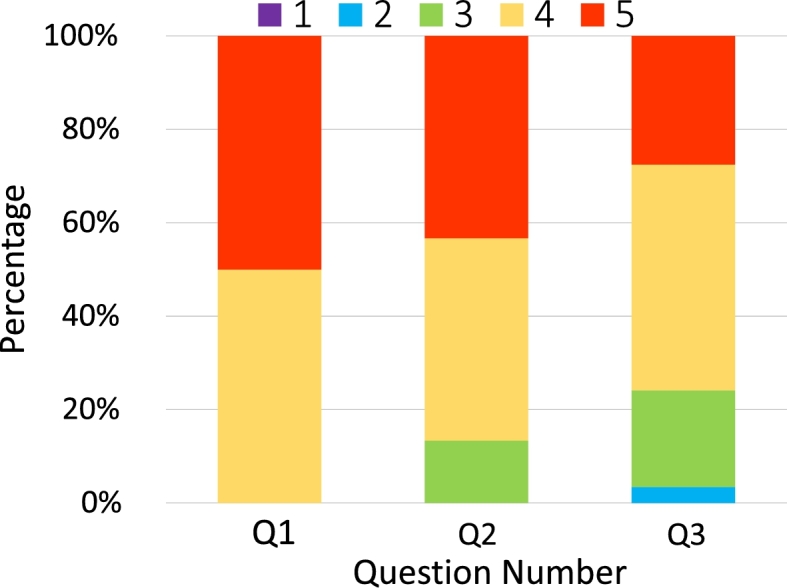
Results of Q1–3 (for RQ2 and RQ3).

**Figure 14:**
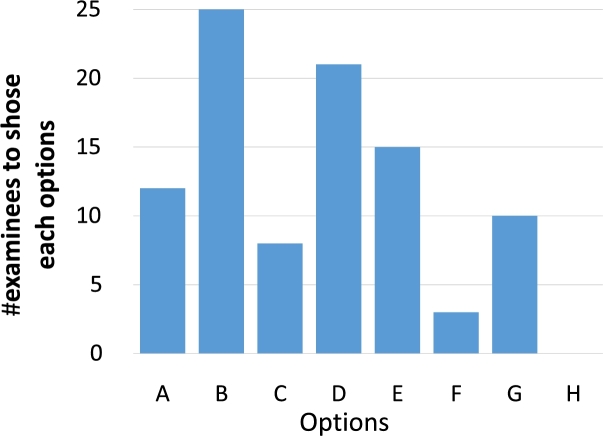
Result of Q4 (for RQ3).

Fig. 11 shows a box plot of the time taken to answer each task, while Fig. 12 shows a bar graph of the percentage of correct answers for the tasks. Figs. 13 and 14 show the questionnaire responses. Fig. 13 shows the participants' answers to Q1–3, which evaluate the usefulness and accessibility of a tool, whereas Fig. 14 shows the answers to Q4, which assesses the utility of PVC. In other words, Fig. 13 represents a measure of the usefulness and accessibility of the PVC program (Q1–3), while Fig. 14 relates the appropriateness of the tool to the application (Q4).

**RQ1**: Figs. 11 and 12 clearly indicate that the group using PVC generally answered the questions more accurately and faster than the other groups. Table 4, which compares the mean values of these results, shows that the group using PVC answered the questions on average 1.8 times faster and gave 24% more correct responses than the group working without a tool (no tool). Compared to the group working with SeeC, those working with PVC responded 1.7 times faster and provided 19% more correct answers, demonstrating that PVC is well suited to the tasks.

**Table 4:** Comparison of PVC, SeeC, and no tool in terms of median, mean, and statistical test.

		Number of correct answers	Time to answer [s]
Mean	no tool	2.5	1148.4
	SeeC	2.1	1045.5
	PVC	3.1	623.3

Median	no tool	3	1002.5
	SeeC	2	1030
	PVC	3	620.5

Steel-Dwass test (p-value)	PVC / no tool	<0.1	<0.01
	PVC / SeeC	<0.05	<0.05
	no tool / SeeC	n.s.	n.s.

Table 4 also shows the *p*-values between the groups and evaluates them using the Steel-Dwass test [26].^8^

A significant difference exists between the groups, except for the relationship between the SeeC and the no tool groups.

Focusing on the results of T3 and T4 of the SeeC group, the correct answer rates are worse than the no tool group, which was unexpected. Moreover, for T3, the SeeC group took more time on average to answer than the no tool group. We speculate that the reasons for these are as follows: T3 — SeeC does not fully support dynamic memory allocation. Thus, participants become confused or have to switch to working with pen and paper, which is more time-consuming. T4 — PVC can visualize the depth of recursive call as a number, but SeeC cannot. This may lead to the difference in the correct answer rate.

Moreover, the participants felt that PVC is very useful for solving the problems in the experiment (Q1). (As shown in Fig. 13, all the participants responded with rankings of 4 or 5.) These results indicate that PVC can convey the status of running programs to programmers in general.

**RQ2**: About 90% of the participants feel that PVC is useful for novices learning some concepts of C programming language (Fig. 13, Q2). As expected, most users felt that PVC is a useful aid for learning pointer and dynamic memory allocation (Fig. 14, Q4). Additionally, participants felt that the program is also useful for functions. Based on these results, we speculate that PVC is useful for checking the answer to T2, which is challenging as it involves a recursive function. Given that task T2 yielded the lowest percentage of correct answers, it is inferred that it is the most challenging part of the experiment.

**RQ3**: About 80% of the participants felt that PVC is more accessible than other existing visualization or debugging tools (Fig. 13, Q3).

Hence, the experiment confirms that PVC mostly satisfies RQ 1–3 proposed at the beginning of the study.

### 5.2 Second experiment

#### 5.2.1 Experimental setting

We conducted a second experiment to answer RQ4 and reconfirm RQ1 and RQ3 by comparing our visualization tool with Python Tutor (PT) and Visual Studio (VS).^9^

PT is a state-of-the-art tool, and PVC.js is inspired by its visualization. VS is a representative traditional visualization tool. VS is one of the most popular IDEs used to debug and visualize the execution status for C language. This experiment used PLIVET instead of PVC.js. PLIVET is the successor to PVC.js (like a version 2), which aims to support other languages (Java and Python). Its function, usage, and visualization for C language on the browser are exactly the same as or slightly better than PVC.js (e.g., the blurred text in the visualization area is clearer in PLIVET)^10^

The participants were 35 university students with at least one earned C language class credit. Eleven performed the tasks using PLIVET, 12 using VS, and the remaining 12 using PT. After completing the tasks using PLIVET, the participants answered a *User Experience Questionnaire (UEQ)*, which is a standard and reliable questionnaire to measure the user experience of interactive products. UEQ consists of 26 questions and classifies the questions into the following six scales: Attractiveness, Perspicuity, efficiency, Dependability, Stimulation, and Novelty.

The experiment involved four tasks that were exactly the same as the first experiment. The participants using PLIVET or PT executed and visualized the program with the few simple buttons provided by the tool. VS is more than a visualization tool, it has many features. However, the participants used only basic debugging commands such as *Execute with Debugging*, *Step Over*, *Step In*, and *Step Out*. VS visualizes the variable name, values, and type at that step in the “Local” window.

**Figure 15:**
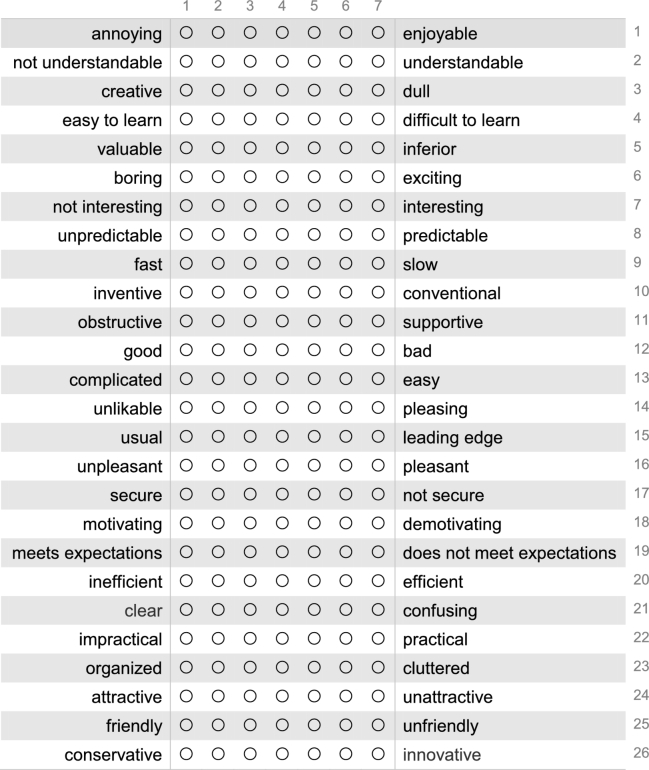
Twenty-six questions of the User Experience Questionnaire.

Fig. 15 shows the questions (Q1–26) in the UEQ. The participants completed the questionnaire upon finishing the tasks using PLIVET. Q1 – Q26 were evaluated on a scale from 1 to 7. Details of this questionnaire are shown elsewhere.^11^

In this experiment, we recorded video on the operation of PLIVET while the participants solved the tasks. Additionally, we recorded the UI interactions of the participants such as the orders of mouse click, the number of clicks, and time of each step.^12^

#### 5.2.2 Experimental results

**Figure 16:**
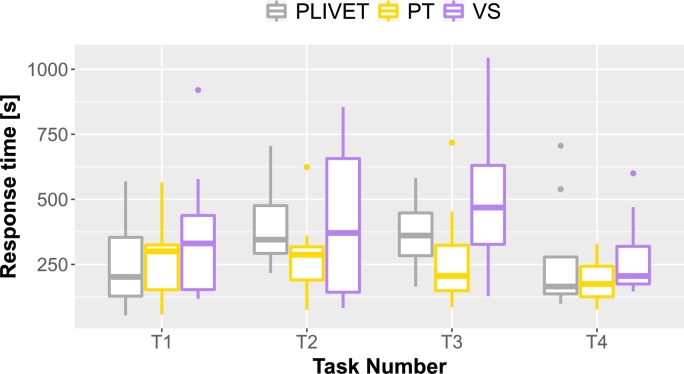
Box plot of the time required to complete each task.

**Figure 17:**
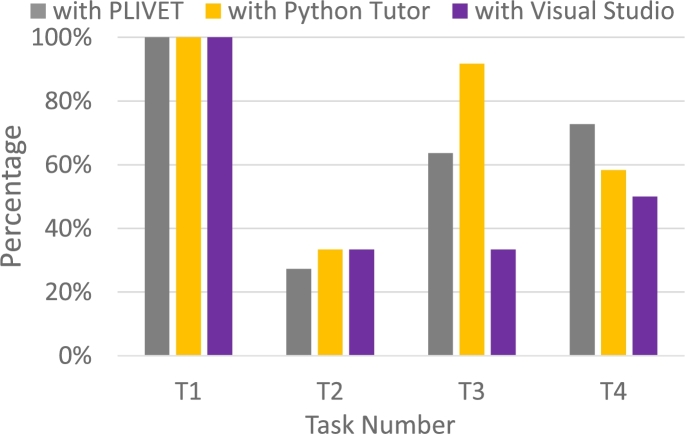
Percentages of correct answers for T1–T4 (for RQ1).

Fig. 16 shows a box plot of the time taken to answer each task, while Fig. 17 shows a bar graph of the percentage of correct answers for the tasks. Table 5 shows the participants' answers to Q1–26 of UEQ, which is a measure of the user experience of PLIVET.

**Table 5:** Results of the UEQ. Scale from 1 to 7 is converted from –3 to +3.

Q	Mean	Left	Right	Scale
1	1.00	annoying	enjoyable	Attractiveness
2	0.45	not understandable	understandable	Perspicuity
3	1.00	creative	dull	Novelty
4	1.82	easy to learn	difficult to learn	Perspicuity
5	2.00	valuable	inferior	Stimulation
6	0.82	boring	exciting	Stimulation
7	0.82	not interesting	interesting	Stimulation
8	1.18	unpredictable	predictable	Dependability
9	1.18	fast	slow	Efficiency
10	1.27	inventive	conventional	Novelty
11	2.27	obstructive	supportive	Dependability
12	1.73	good	bad	Attractiveness
13	-0.09	complicated	easy	Perspicuity
14	1.36	unlikable	pleasing	Attractiveness
15	-0.09	usual	leading edge	Novelty
16	0.73	unpleasant	pleasant	Attractiveness
17	1.45	secure	not secure	Dependability
18	1.27	motivating	demotivating	Stimulation
19	1.18	meets expectations	does not meet expectations	Dependability
20	1.09	inefficient	efficient	Efficiency
21	1.09	clear	confusing	Perspicuity
22	3.00	impractical	practical	Efficiency
23	0.82	organized	cluttered	Efficiency
24	1.36	attractive	unattractive	Attractiveness
25	1.36	friendly	unfriendly	Attractiveness
26	0.27	conservative	innovative	Novelty

**RQ1**: Figs. 16 and 17 indicate that there is not much difference between PLIVET and PT. Both are slightly better than VS. Table 6, which compares the mean values of these results, shows that the group using PLIVET answered the questions on average 1.3 times faster and gave 18% more correct responses than the group working with VS. However, according to the *p*-values, a significant difference does not exist between the PLIVET and the other tools. These results reaffirm the premise of the first experiment, which indicated that our tool and PT have similar visualization performances.^13^

**Table 6:** Comparison of PLIVET, VS, and PT in terms of median, mean, and statistical test.

		Number of correct answers	Time to answer [s]
Mean	PLIVET	2.6	1250.4
	VS	2.2	1566.4
	PT	2.8	1007.6

Median	PLIVET	3	1110
	VS	2	1614.5
	PT	3	963.5

Steel-Dwass test (p-value)	PLIVET / VS	n.s.	n.s.
	PLIVET / PT	n.s.	n.s.
	VS / PT	n.s.	<0.1

**RQ3**: Table 7 shows the mean of the UEQ scales for the tools. PLIVET has good efficiency and stimulation. On the other hand, Perspicuity and Novelty are inferior to the others, but received positive evaluations. At all scales, PLIVET and PT show higher points than VS, which represents existing tools. Table 8 shows the statistical significance of the UEQ scales for the tools. There is not a significant difference between PLIVET and PT, but there is a significant difference with VS.

**Table 7:** Results of the UEQ as the six UEQ scales.

UEQ Scales	Mean		
	PLIVET	PT	VS
Attractiveness	1.26	2.04	0.50
Perspicuity	0.82	1.44	0.08
Efficiency	1.52	1.15	0.88
Dependability	1.52	1.40	0.81
Stimulation	1.23	1.96	0.81
Novelty	0.61	0.85	0.00

**Table 8:** Steel-Dwass test result (p-value) of the UEQ scales.

	Attractiveness	Perspicuity	Efficiency	Dependability	Stimulation	Novelty
PLIVET / VS	n.s.	n.s.	n.s.	<0.1	n.s.	n.s.
PLIVET / PT	n.s.	n.s.	n.s.	n.s.	n.s.	n.s.
VS / PT	<0.01	<0.05	n.s.	n.s.	<0.01	n.s.

Hence, the experiments confirm that PLIVET (PVC.js) is equivalent to the advanced tool PT and it has better visualization capabilities than the existing tool VS. These finding make the answer to RQ1 in the first experiment more robust.

**Figure:**
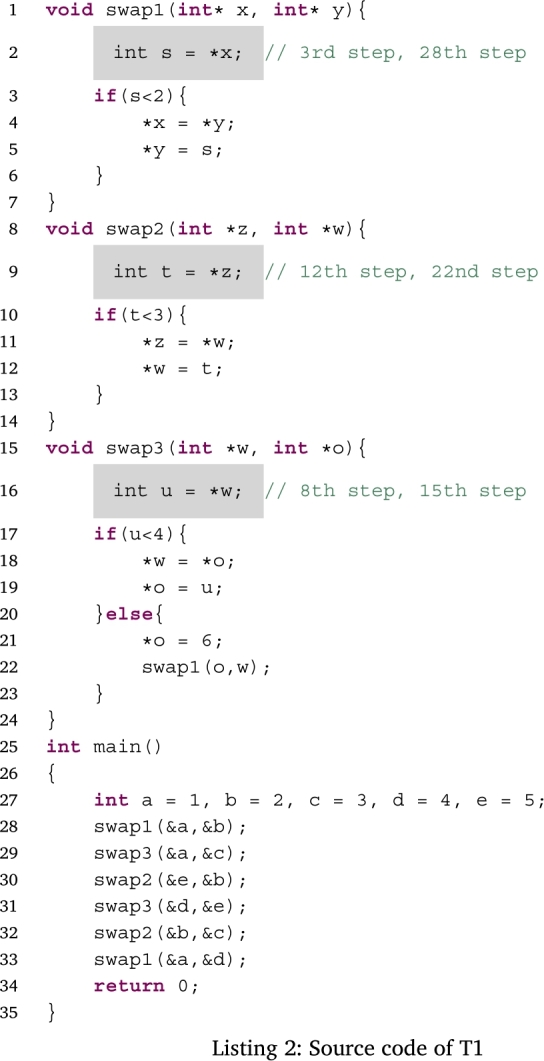

**Figure:**
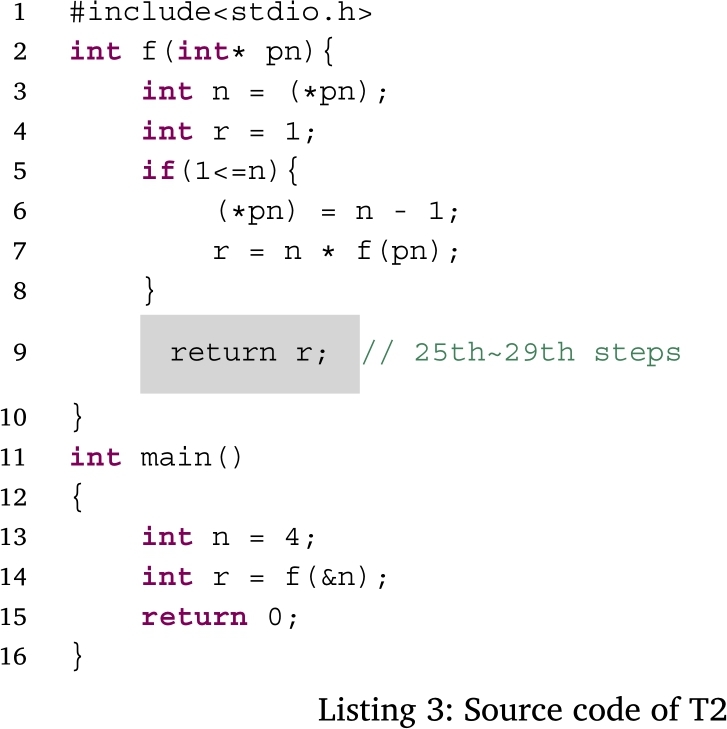

**Figure:**
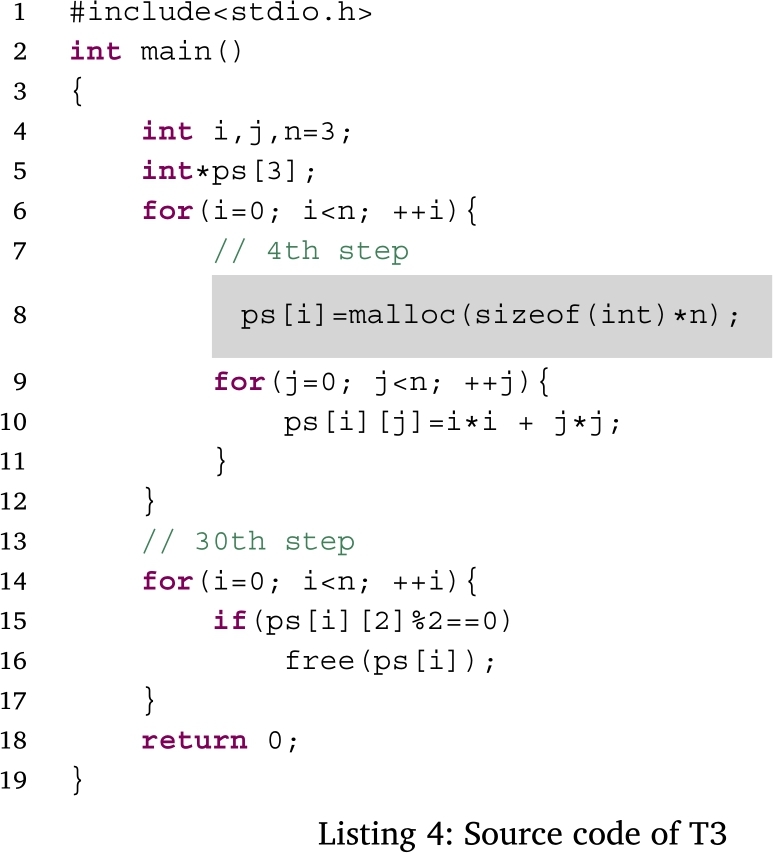

**Figure:**
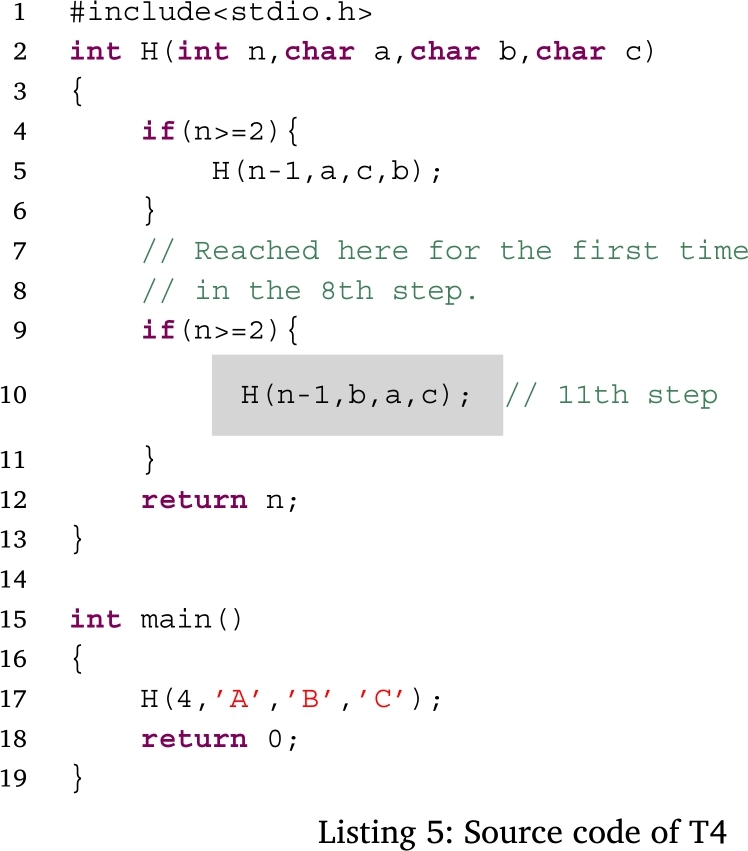

**RQ4**: Table 9 shows the average number of times that the execution controller button was clicked in each task. Once the participants clicked *the button to initiate program execution*, they basically used *the button to go forward one step*.

**Table 9:** Average number of times each button was clicked during a task (T1–4).

Controller buttons	T1	T2	T3	T4
Initiate program execution	1.3	1	1	1.2
Stop program execution	0.1	0	0	0.3
Go backward for all step	0.2	0.5	0.2	0.9
Go backward one step	0.8	23	21.8	22
Go forward one step	27.9	79.9	81	82
Go forward all steps	0.5	0.2	0.3	0

**Figure 18:**
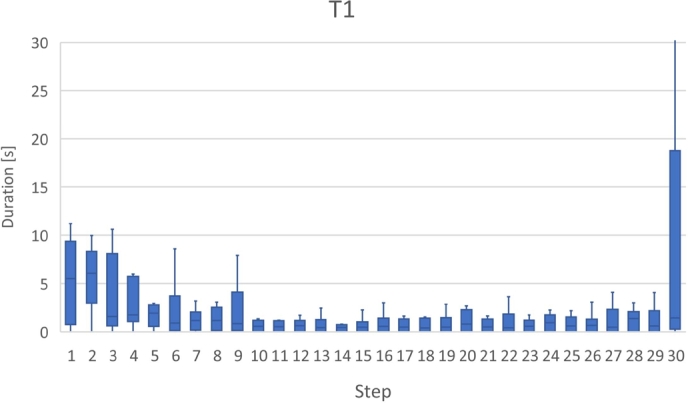
Duration of each step in T1.

**Figure 19:**
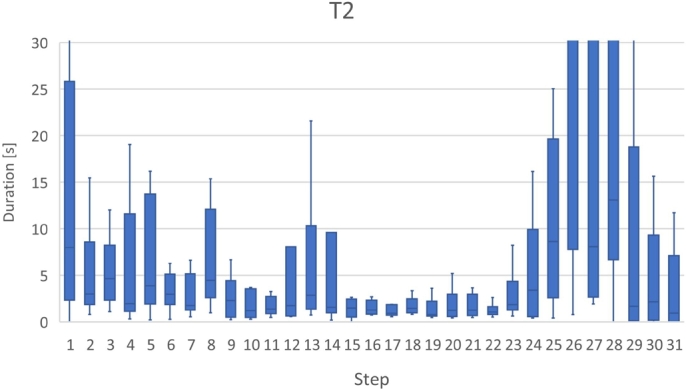
Duration of each step in T2.

**Figure 20:**
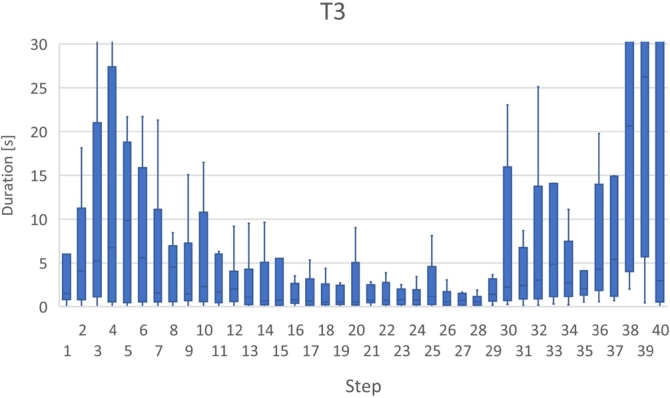
Duration of each step in T3.

**Figure 21:**
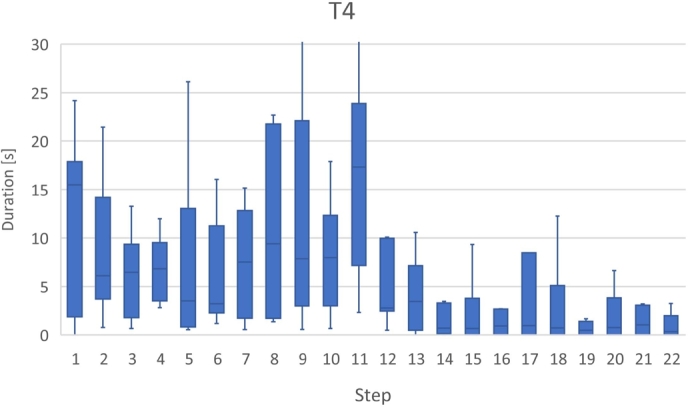
Duration of each step in T4.

Figs. 18, 19, 20, and 21 show the time that the participants stayed at the n-th step of the program for each task. The horizontal axis shows the n-th step of the program. The vertical axis shows the number of seconds the participants stayed at each step. For simplicity, the y-axis is displayed in the range of 30 seconds or less.

For T1 (Listing 2), *the button to go forward one step* was used after clicking *the button to initiate program execution*. The participants stayed for only a few seconds in most steps. However, they stayed longer in the 1st–3rd steps, 6th step, 9th step, and 30th steps. The 1st–3rd steps defined five new variables and the swap function was called for the first time. In the 6th step, processing returned from the swap1 function to the main function. In the 9th step, a value was overwritten by another value. In the 30th step, all function calls were finished and the answer values were visualized. Thus, we expect that the participants were trying to understand the program fully in steps where function calls and the value of a variable changed.

For T2 (Listing 3), the participants stayed for a long time after the 25th step. This may be because the recursive function was returned in these steps and the answer values were also visualized. All the participants used *the button to go backward one step* several times around the 27th step. Hence, it is considered that the participants tried to understand the program by seeing the difference in the visualization result at each step.

For T3 (Listing 4), the participants spent a lot of time in the 4th and 29th–33th steps. In the 4th step, the value was assigned to the heap area allocated by the *malloc* function. The heap area was released with the *free* function around the 34th step.

For T4 (Listing 5), the participants spent a long time in the 8th–11th steps. Before the 12th step, the first *H* whose arguments were *(n-1,a,c,b)* in the function *H* was called recursively and consecutively four times. After that, the second *H* whose arguments are *(n-1,b,a,c)* in the function *H* called in the function *H* called in the third time. The participants also used *the button to go backward one step* several times to understand the program because the control flow was fairly complicated. Moreover, the participants used *the go backward for all step button* most frequently in this task. According to this result, it is speculated that the participants tried to deepen their understanding of the program by repeating the execution of the program.

Hence, we found that PVC.js supports the understanding of the program by the step forward and back execution functions, especially in cases where the values of important variables change and the control flow is complicated. This is answer to RQ4.

### 5.3 Threats to validity

All the participants in this study had basic knowledge of the C language and we provided a tutorial of PVC.js. However, the degree of proficiency varied within the groups. Some participants used PVC.js very well, while others struggled in the experiment. These attributes may affect the results of the experiment and pose a threat to internal validity.

Most participants were students of Waseda University and Osaka Institute of Technology. If we repeated this experiment with people belonging to another group or organization, the results may vary. The results may depend on demographics (e.g., age), programming skill level, and experience in the field. These pose a threat to the experiment's external validity.

### 5.4 Limitations

Currently, PVC.js supports most C90 keywords, but it does not support minor language features. Table 10 shows the keywords supported by current PVC.js.^14^ For example, it does not support *union* and external libraries. Other tools such as SeeC and PT support all keywords up to C11 because they use major compilers such as *gcc*. However, PVC.js is designed for novices. We believe that the lack of advanced C features is acceptable as advanced features are unnecessary for novices to learn programming. On the other hand, C98 features are partially supported, such as *bool* type and variable declarations in the middle of function blocks. C11 and C17 features are not supported.

**Table 10:** C Language keywords supported by the current PVC.js.

C Keywords	support (Y/N)
auto	N
break	Y
case	Y
char	Y
const	Y
continue	Y
default	Y
do	Y
double	Y
else	Y
enum	N
extern	ignored
float	Y
for	Y
goto	N
if	Y
int	Y
long	Y
register	N
return	Y
short	Y
signed	Y
sizeof	Y
static	N
struct	Y
switch	Y
typedef	Y
union	N
unsigned	Y
void	Y
volatile	ignored
while	Y
_Bool (C99)	Y (as bool)
inline (C99)	ignored
restrict (C99)	N
_Complex (C99)	N
_Imaginary (C99)	N
_Alignas (C11)	N
_Alignof (C11)	N
_Atomic (C11)	N
_Generic (C11)	N
_Noreturn (C11)	N
_Static_assert (C11)	N
_Thread_local (C11)	N

Moreover, PVC.js does not display compiler error messages because it is a visualization tool and not a compiler.

## 6 Conclusions and future work

We propose a new visualization technique for C languages called PVC.js. It is a browser-based JavaScript application inspired by previous studies. The application is open-access and free to try at the listed address (see Section 1). The experiment reveals that PVC.js is useful not only to novices, but also to programmers in general.

In the future, we plan to investigate whether PVC.js can help students learn programming by evaluating users' programming skills after using PVC.js. Moreover, we will continue to develop and improve PVC.js. For example, our visualize application currently supports only C language. It does not support the full range of C language syntax and standard library functions. We are also planning to improve PVC.js to increase the support functions, support multiple programming languages and incorporate some suitable block-based representation (e.g. **blockly**^15^ can transpile blocks to code). Such block-based representation can make it easier for the learners to avoid using not supported features and also protect them from syntax errors.

## Declarations

### Author contribution statement

R. Ishizue, K. Sakamoto: Conceived and designed the experiments; Performed the experiments; Analyzed and interpreted the data; Contributed reagents, materials, analysis tools or data; Wrote the paper.

H. Washizaki, Y. Fukazawa: Conceived and designed the experiments; Analyzed and interpreted the data.

### Funding statement

This work was partially supported by Japan Science and Technology Agency (JST) Presto under Grant Number JPMJPR14D4.

### Competing interest statement

The authors declare no conflict of interest.

### Additional information

No additional information is available for this paper.

## Footnotes

1 This paper is an extension a poster “An Interactive Web Application Visualizing Memory Space For Novice C Programmers” [6] presented at the 48th ACM Technical Symposium on Computer Science Education (SIGCSE 2017) and a paper “PVC: Visualizing C Programs on Web Browsers for Novices” [7] presented at the 49th ACM Technical Symposium on Computer Science Education (SIGCSE 2018). In the previous papers, we solved the problem of installability by using a local Java server in an offline environment. In this paper, we develop a new JavaScript application called PVC.js, which does not require a web server to improve S2. We also provide a more detailed description of the application, conduct another experiment to answer an additional research question, add a detailed description of each experimental task, and compare additional related works. In summary, we propose a new tool, which is easier for novices to use, conduct an additional experiment, and explain the important concepts about tools and experiments in more detail.

2 A **novice** is defined as a person learning C language who is currently trying to understand the concept of memory management, including pointers and dynamic memory allocation.

3 Github of PT says “it can be hard to run your own visualizer locally for non-Python languages, since there are complex setups in v4-cokapi/ that I haven't yet cleanly packaged up.”

5 PVC is published as a web application. Its function, usage, and display on the browser are exactly the same as PVC.js.

6 For the above reason, we judged that there is no visualization difference between PT and SeeC. Hence, this experiment only employed SeeC.

7 http://play-visualizer-c.herokuapp.com.

8 We selected the Steel-Dwass test, which is a non-parametric test, because it is difficult to determine whether the scores and times of the tasks follow a normal distribution that strictly avoids multiple test.

9 The first experiment revealed the difference between PVC.js and SeeC. Hence, SeeC is excluded in this experiment.

10 https://github.com/RYOSKATE/PLIVET.

11 https://www.ueq-online.org.

12 Since one participant's recorded data was incomplete, 10 participant's data were used.

13 The time to answer and the number of correct answers are lower than in the first experiment because the second experiment targeted a group with more beginners.

14 PVC.js is evolving (as PLIVET). The latest supported C keywords can be found at https://github.com/RYOSKATE/PLIVET/wiki.

15 https://developers.google.com/blockly.

## Footnotes

4 Dynamically allocated memory can be visualized if *sizeof(type)* is used in the arguments of *malloc*.
